# Editorial: Integrated systems genomic approaches for characterizing uncharacterized proteins

**DOI:** 10.3389/fgene.2022.1000825

**Published:** 2022-09-13

**Authors:** Jayaraman Valadi, Vijayaraghava Seshadri Sundararajan, Obul Reddy Bandapalli, Alfredo Benso, Prashanth Suravajhala

**Affiliations:** ^1^ Department of Computer Science, Vidyashilp University, Maharastra, India; ^2^ Bioclues.org, Hyderabad, India; ^3^ Department of Computer Science, FLAME University, Pune, India; ^4^ PolitoBIOMed Lab—Biomedical Engineering Lab, Turin, Italy; ^5^ Amrita School of Biotechnology, Amrita Vishwa Vidyapeetham University, Kollam, Kerala, India

**Keywords:** known unknowns, hypothetical proteins, systems genomics, bioinformatics, annotation

The machinery of life is made possible by proteins. Each protein is characterized by its unique amino acid sequence which determines its shape as well as its function. Over the years, researchers have shown how the shape and functions of relatively small numbers of proteins vary (Adams, 2008) from which functions of these proteins could be ascertained. On the other hand, there are known unknown regions in the genome which are the uncharacterized sequences. The genes which are known but whose functions are not known are called orphan genes and their corresponding protein products are called hypothetical proteins (HPs; Galperin, 2011; Suravajhala and Sundararajan, 2012). In this special issue, authors show how characterizing the uncharacterized sequences can advance the state of the art in different fields, from studying drug resistance in Malaria, to understanding biochemical mechanisms that allow them to adapt to cold-habitats besides investigating bacterial antibiotic resistance using the HPs. The topic entails four articles integrating *bona fide* computational tools for discerning the functions of the known unknown regions in the genome.

Singh and Gupta describe annotation of *Plasmodium falciparum* HPs with an aim to understand the growing drug resistance in Malaria, which, over the years has created an urgent requirement for alternative and more effective antimalarial drugs or vaccines (Singh and Gupta). The availability of complete genomes of various Plasmodium species has opened new opportunities for development of drugs and vaccines, albeit with mixed success. This, the authors, emphasize results in a poor understanding of the parasite biology and its incomplete functional annotation. While the Plasmodium genomes are yet to be completely annotated with exact mechanisms of pathogenesis in human hosts, a host of proteins are currently not annotated. For example, of 5,389 proteins in Plasmodium falciparum 3D7 strain 1,626 proteins (∼30% data) are labeled as “hypothetical”. Annotation of these HPs may lead to better understanding of the parasite mechanisms to evade and exploit host anti parasitic mechanisms. To bring the work closer, they employ a computational pipeline through which they identify 266 conserved functional signatures with essential candidates attributing to various biochemical, signaling and metabolic pathways. Furthermore, they could successfully model 11 proteins and perform molecular dynamic simulations to check for stable conformations. Thus, they could deploy a protein-protein interaction (PPI) network revealing 3,299 nodes and 2,750,692 edges. The work attempts to bridge the gap for experimenting candidate HPs (Singh and Gupta).

Ijaq et al. discuss the importance of studying the molecular mechanisms emphasizing the adaptation of a cold-habitat Gram-negative psychrophilic Antarctic bacterium *Pseudomonas spp. Lz4W* that can survive in extreme temperature conditions. The authors used explorative proteome analyses to identify the proteins and pathways that might be aiding for the adaptation and survival of the bacteria. By employing a bioinformatics pipeline which they describe, they studied that Pseudomonas sp. Lz4W genome (CP017432, version 1) which were found to contain 4,493 genes, 4,412 coding sequences (CDS), of which 743 CDS were annotated as HPs. Among them, 61 HPs were found to be expressed consistently at the protein level with 18 functionally assigned which were found to be involved in peptidoglycan metabolism, cell wall organization, ATP hydrolysis, outer membrane fluidity, catalysis aiding in the biological mechanisms of cold adaptations. Their study aimed to provide a deeper understanding of the functional significance of HPs in cold adaptation of Pseudomonas sp. Lz4W. The authors further submitted the data generated from MS proteomics to the ProteomeXchange through PRIDE (PXD029741; Ijaq et al.).

Liu et al. studied the role of HPs in *Mycobacterium tuberculosis* and BCG strains with a focus on the mortality rate associated with Tuberculosis. The authors perform a comparative proteomic profiling of intracellular and extracellular virulent strain M. tb and bacille Calmette–Guérin (BCG) from infected THP-1 cells through incubation and post incubation studies. They identify a total of 1,557 proteins, which were divided into four groups for comparison of M. tb versus BCG under 7H9 culture, M. tb one-type versus M. tb second-type, BCG one-type versus BCG second-type, and other combinations. Among them, 8 proteins were found to be associated with ESAT6, related to virulence besides 5 uncharacterized proteins that were differentially expressed. Further, pathway enrichment analysis revealed that glyoxylate and dicarboxylate metabolism pathways were majorly involved in the biological process and these findings, the authors claim to have valuable data for further exploration of molecular mechanisms for M. tb virulence and BCG immune response (Liu et al.).

Bhat et al., reviewed in silico approaches in the form of bioinformatics. They discussed the applications of bioinformatics in collecting the experimental data, organizing them in several databases available online like nucleotide database, protein databases, GenBank and others and employing them as reference for experimental evaluation and validation in the form of genomic, transcriptomic, proteomics, epigenomics and metabolomics data. The application of characterizing the uncharacterized proteins include the discovery of new metabolic pathways which can be targeted for therapeutic purposes, development of vaccines, and novel potential drug targets for drug discovery. They further describe mutational databases and regulatory genome databases, including Human Splicing Finder (HSF), Exonic Splicing Enhancer Mutation taster, aiding in better understanding of the intronic mutations that can be further validated experimentally (Bhat et al.).

Abbasi et al. provided an *in silico* approach to characterize the HPs of six different *Clostridium difficile* strains. A multi-strain, spore-forming, Gram-positive, opportunistic enteropathogen bacteria, *Clostridium difficile* (*C. difficile*) is primarily linked to nosocomial infections, which cause severe diarrhea and colon inflammation. The report suggests that the excessive use of antibiotics has led to the development of antibiotic resistance in *C. difficile* strains. The authors have emphasized the need for functional annotation of the HPs that were found in the genome of *Clostridium difficile* using various bioinformatics tools in which they analyzed complete genomes, including contigs identification, coding sequences, phages, CRISPR-Cas9 systems, antimicrobial resistance determination, membrane helices, instability index, secretory nature, conserved domain, and determined its physicochemical properties along with vaccine target properties like comparative homology analysis, allergenicity, antigenicity determination along with structure prediction and binding-site analysis to annotate its possible functions in vaccine development and potential drug targets. They identified ca. 32–38% and 23–27% of HPs were localized in the cytoplasm and cytoplasmic membrane, respectively. While 99.7% of the identified HPs across all the selected strains were non-homologous to humans, and show no virulence factors, they further exploited the antigenicity of the HPs and successfully model highly antigenic proteins using structure prediction and their binding sites. They underpin the most recent developments in the adage of next generation sequencing (NGS) with vivid curation tools which help understand and discover novel drugs for ascertaining the known unknown regions in bacteria (Abbasi et al.).

In the realm of biology, the known unknown regions (Logan, 2009) could be better ascertained using hypothome (∼ an interactome of hypothetical proteins, which we proposed earlier; Desler et al., 2014) with multidisciplinary subjects such as systems genomics, bioinformatics aided functional genomics and third generation sequencing technologies with a focal interest on identifying candidate regions associated with causality. There is a room for exploring more tools and fundamental processes elucidating the mechanisms underpinning small molecular interactions and deploying methods for ascertaining the function of HPs. In conclusion, multi-integration is the key challenge.
